# Social Sustainability of a Firm: Orientation, Practices, and Performances

**DOI:** 10.3390/ijerph192013391

**Published:** 2022-10-17

**Authors:** Xiaozhen Wang, Mark Yang, Kihyun Park, Ki-Hyun Um, Mingu Kang

**Affiliations:** 1 School of Innovation and Entrepreneurship, Zhejiang University of Science & Technology, Hangzhou 310023, China; 2 Department of Management, College of Business and Public Management, West Chester University, West Chester, PA 19383, USA; 3 Department of Management, Rockwell School of Business, Robert Morris University, Moon, PA 15108, USA; 4 College of Business Administration, Pukyong National University, Busan 48513, Korea; 5 School of Management, Zhejiang University, Hangzhou 310058, China

**Keywords:** social sustainability, social exchange theory, social identity theory, employee well-being and equity, corporate social involvement

## Abstract

This paper investigates how firms’ social sustainability practices can influence their social performance and, ultimately, financial performance. We include two corporate social sustainability practices: employee-oriented (employee well-being and equity) and socially driven (corporate social involvement) practices. Three leading social theories (social identity theory, social exchange theory, and resource-based view) are applied in explaining how firms’ social practices influence intermediate and bottom-line performance outcomes. Empirical results of 212 US manufacturing firms reveal that (1) the social orientation of the firm promotes firms’ social performances (employee-oriented and community-oriented outcomes) directly; (2) social orientation also indirectly promotes employee-oriented outcomes via employee well-being and equity practices, and so does community-oriented outcome via corporate social involvement practices; and (3) the firms’ social performances can enhance financial performance. The theoretical and managerial implications derived from these empirical results are discussed as well.

## 1. Introduction

Over the past decades, sustainability and sustainable development have gained much attention among academic scholars and practitioners. The interrelationships between society, environment, and economic development are key to the concept of sustainability [1,2]. The literature on sustainability highlights the importance of equally satisfying economic, social, and environmental priorities to ensure comprehensive sustainable development [3,4]. The economic dimension of sustainability often covers how to improve firms’ operational, market, and financial performances [5], while the environmental dimension of sustainability focuses on consuming less natural resources, with the goal of preserving the natural environment [4]. Specifically, the concept of social sustainability is somewhat similar to corporate social responsibility. However, there are also some distinctions between them. For instance, different from corporate social responsibility, social sustainability excludes “attention to other types of unethical, irresponsible business practices from accepting slavery in production to perpetrating fraud on the consumer” [6] (p. 13). In this way, all socially responsible practices are not necessarily socially sustainable [7]. Sheehy and Farneti [6] also argue that the terms corporate social responsibility, corporate sustainability, sustainability, and sustainable development have areas of overlap and also reveal differences, especially when identifying their distinct policy objectives and policy scope.

In the literature, the concept of the social dimension of sustainability is often addressed in a vague and inconsistent manner, thus leading to multiple concepts due to different aspects [2,8]. For instance, Eizenberg and Jabareen [2] proposed a conceptual framework of social sustainability with the vision of having a safer planet and suggested four interrelated concepts of socially oriented practices, such as safety, equity, physical urban forms, and eco-prosumption. Hutchins and Sutherland [1] also suggested indicators of social sustainability on a national scale by focusing on human health, safety, equity, and quality of life. At an organizational and supply chain level, Hutchins and Sutherland [1] proposed indicators for the social dimension of sustainability, including employee factors (i.e., labor equity, healthcare, and safety) and philanthropic roles within a community and to society at large. Similarly, according to Steurer et al. [9], the social dimension of sustainability considers both internal social improvements for stakeholder group employees and external social improvements for a variety of other stakeholder groups such as communities or neighborhoods and supply chain partners. First of all, the social dimension of sustainability aims at enhancing employee well-being (or health and safety) and human rights (or equity) [10]. Second, it is based on being a responsible organization toward society or community [11]. Since the current study focuses on manufacturing firms’ social sustainability practices, we focus on both internal (employee) and external (community and society) aspects of firms’ social responsibility. In other words, social sustainability practices are defined as the extent to which an organization implements plans/programs to improve its employee and communal performance.

There is a large body of literature that looks at how corporate sustainability practices might improve firm performance [12,13,14,15]. However, previous literature has focused more on sustainability’s economic and environmental aspects when examining the relationship between sustainability and performance outcomes [7]. Little research has investigated social sustainability practices’ strategic role in promoting firms’ financial performance. Without concrete evidence of directly influencing financial performance, firms with a clear purpose and noble goals to implement social sustainability practices can barely establish a socially responsible corporate culture. Specifically, there are research gaps in the literature concerning the underlying mechanisms by which social sustainability practices influence firms’ financial performance. In explaining how firms’ social sustainability practices can lead to better performance outcomes, we apply social identity theory (SIT), social exchange theory (SET), and resource-based view (RBV). Social identity theory suggests that firms’ social sustainability engagement in social good will positively enhance employees’ perceived image or identity of a company, encouraging employees’ better commitment to the company and increasing firms’ performance. Social exchange theory supports that firms’ proactive orientation on employees, such as enhancing their safety and well-being, will positively reinforce employees’ attitudes and behavior toward their companies. The resource-based view assumes that firms create a competitive advantage by developing new capabilities and differential abilities related to socially sustainable performance. Based on these theories, this study suggests a research model and attempts to answer the following two research questions: (1) Will firms with greater social orientation (i.e., highly employee-oriented and socially driven firms) bring positive social sustainability practices? (2) How do two dimensions of social sustainability practices influence social sustainability performance and financial performance? By answering these questions, this study provides valuable insights into how firms’ social orientation leads to better social and financial performance through social sustainability practices.

## 2. Literature Background

### 2.1. The Social Orientation (SCO) of the Firm

A firm’s social orientation (SCO) can be considered a subset of the firm’s strategic orientation toward sustainability. In their early work, Pagell and Gobeli [10] defined managerial sustainability orientation as the extent to which an organization is proactive and committed to economic, environmental, and social concerns in its decision-making across its supply chain. To support this overarching concept, they included the conceptual model of strategic sustainability orientation in the following ways. First, economic sustainability should be aligned with the firm’s environmental and social goals. Second, sustainability should be a part of a firm’s day-to-day conversation, not an add-on or afterthought in decision-making. Third, a core value that guides a firm’s business model should be aligned with a nontraditional element of sustainability across all employees and functions. The firm’s responsible orientation toward social and environmental aspects of sustainability is often highlighted as sustainability orientation in the literature.

Marz et al. [16] tackled an interesting topic on how corporate (such as hierarchy level and professional activities) and individual characteristics (such as political system origin and gender) influence managers’ SCO. Smith et al. [17] tested how corporate SCO influences firms’ attractiveness in adopting affirmative action programs versus diversity programs. Brickson [18] developed the framework of organizational identity orientation concerning its stakeholders, such as customers, nonprofits, and employees. Burton and Goldsby [19] explored how corporate social responsibility orientation is related to managers’ behaviors and goals. The results show that business owners’ behavior is directly affected by their attitudes toward sustainability. Bingham et al. [20] argued that how much firms engage in corporate social performance (CSP) is caused by the firm’s stakeholder identity orientation. Their finding suggests that firms react to social activities differently based on their identity orientation. One recent study examined students’ SCO in higher education [21]. Most studies, however, largely miss how firms’ SCO affects the actual practices. In this study, social SCO is defined as the extent to which a firm is proactive and committed to positive employee and communal priorities in its decision-making.

### 2.2. Social Sustainability Practices

First, employee well-being and equity practices (EWEP) are one of the key internal factors of social sustainability practices. ISO 26000 standards were introduced to address social sustainability concerns effectively and help companies and their supply chains address those issues [22]. The key components of ISO 26000 are employee-related issues (i.e., human rights and workplace), such as occupational health and safety, unfair business practices, bribery, corruption, anti-competitive practices, organizational governance, and social development. Reflecting such trends, scholars have attempted to measure workplace or employee-related social sustainability practices. For example, Hutchins and Sutherland [1] used labor equity, healthcare, and safety to measure a firm’s social sustainability. Vachon and Mao [23] used women’s employment and wage equality in the workplace to measure a firm’s fair labor practices and social equity. Traditional operation management studies examined employee well-being regarding occupational health and safety [24]. The assumption is that the well-being of employees or workers will be enhanced only when a safe or risk-free working environment is ensured [25]. Sustaining workplace safety has been directly tied to not only safeguarding employees’ health and safety but also promoting their welfare, since work-related injuries and accidents have gradually increased, risking employees’ well-being [26]. Regarding social sustainability, growing attention also has been paid to employee equity, such as fair business practices, protecting human rights, fair compensation, and gender equity [27,28,29]. In this study, we define EWEP as the extent to which a firm promotes and improves the quality of employees’ health/safety and human rights.

Second, another social sustainability practice is corporate social involvement practices (CSIP). Elkins [30] emphasized that firms’ active involvement in the external social issue is a positive phenomenon motivated by corporate morality, protective strategy, managerial ego satisfaction, profit-seeking purpose, and public relations or advertising value. Over the decades, large corporations also put increasing efforts into social or community engagement in the pursuit of being socially responsible firms. For instance, Procter & Gamble, the manufacturer of Pampers disposable diapers, has been actively involved in socially responsible sales-improving practices, such as infant life-saving health programs and education. Companies supporting discretionary responsibilities, such as philanthropic donations and educational programs, seek to become socially legitimate within the larger communities [1]. Based on the previous literature [1,9,23,31], this study considers the concept of CSIP as an external factor of social sustainability practice by focusing on firms’ philanthropic commitment within a community and to society at large. Moreover, it examines the CSIP’s intervening role in the relationship between SCO and community-oriented outcomes.

## 3. Hypotheses Development

### 3.1. Social Orientation and Social Sustainability Practices

Generally, a firm’s sustainability orientation is reported to be important in adopting sustainability practices [32]. A firm’s SCO is considered a subset of the firm’s strategic orientation toward sustainability [33]. Thus, a firm oriented toward social sustainability issues will likely adopt social sustainability practices. The SCO of the firm shows how much firms are dedicated to corporate social responsibility, including employee well-being, equity, and social involvement. Firms with a greater SCO do not remain in their higher strategic thinking. Rather, their social sustainability culture is embedded with their mission and vision, which can be transferred to their operational-level practices [33]. Since the SCO of the firm involves a proactive approach toward employees and society, firms with a greater SCO will enhance the firm’s social sustainability activities that address employee safety and well-being as well as social responsibility. According to Croom et al. [34], social sustainability-oriented firms are deeply rooted in social sustainability values, and thus they are not negligible in their impact on society. Such a value- and purpose-driven propensity will align their decision-making to meet society’s expectations toward employee treatment and contribution to society. When companies are socially responsible, they are likely to adopt social sustainability practices above and beyond regulation. Thus, the following hypotheses are proposed:

**H1a.**  *Firms’ social orientation is positively related to their implementation of employee well-being and equity practices.*

**H1b.**  *Firms’ social orientation is positively related to their implementation of corporate social involvement practices.*

### 3.2. Social Orientation and Social Sustainability Performances

We also argue that a firm’s greater social sustainability orientation leads to the firm’s social sustainability practices and social sustainability performance. Research suggests that firms’ strategic orientation is a powerful predictor of firm performance. Croom, Vidal, Spetic, Marshall, and McCarthy [34] examined how social sustainability orientation impacts firms’ operational performance through socially sustainable supply chain practices. They found that these relationships are highly influenced by a firm’s long-term social sustainability orientation. We argue that socially oriented firms are better positioned to implement social sustainability practices and bring better employee- and community-oriented performance outcomes. Thus, the following hypotheses are proposed:

**H2a.**  *Firms’ social orientation is positively related to their employee-oriented outcomes.*

**H2b.**  *Firms’ social orientation is positively related to their community-oriented outcomes.*

### 3.3. Employee Well-Being and Equity Practices and Employee-Oriented Outcomes

As mentioned above, EWEP refers to a firm improving the quality of employees’ health/safety and human rights. Since the firm makes efforts to provide a safe work environment, fair compensation, and gender equality, employees are more likely to ensure their health and safety and fair compensation and opportunity. In addition, since EWEP may shape the formal or informal organizational ethical climate, managers are more likely to demonstrate fairness and concern about human rights [35]. Therefore, EWEP is more likely to enhance employees’ quality of life and satisfaction. Therefore, we propose that EWEP increase EOO such as employee quality of life, safety, and fair compensation.

**H3.**  *Employee well-being and equity practices are positively associated with employee-oriented outcomes.*

### 3.4. Corporate Social Involvement Practices and Community-Oriented Outcomes

In applying social identity theory to the CSR context, we predict the effect of CSIP on COO. Social identity theory illustrates how individuals identify themselves based on their group membership(s) [36]. When applied in an organizational and managerial context, this theory proposes that employees will identify more strongly with an organization when they believe it to be impeccable because their sense of belonging to that company raises their self-esteem [37,38]. Implementing CSIP is expected to shape employees’ perceptions of the organization’s ethics and social roles, motivating them to create a positive self-esteem [39,40]. The firm expects its employees to be proud of belonging to the organization, bolster their self-esteem, and enhance their identification with that organization, eventually enabling the firm to reach its goals [41,42]. Thus, social involvement practices in organizations encourage employees to show social commitment and contribute to communities, which lead to corporate reputation and closer relationship with the local community. Thus, we predict:

**H4.**  *Corporate social involvement practices are positively associated with community-oriented outcomes.*

### 3.5. Social Sustainability Performances and Financial Performance

In this section, we propose that both EOO and COO positively affect firms’ financial performance. Drawing upon SET and RBV, we consider corporate sustainability outcomes as determinants of bottom-line performance. These frameworks provide theoretical underpinnings that link the interrelationships among SCOs to values and identities, internal and external sustainability outcomes, and business performance. One central tenet of SET is the rule of reciprocity: if one party offers a benefit, the receiving party should reciprocate by providing some benefit in return [43]. Reciprocity is activated when fair returns for the inputs are received (i.e., the rewards are higher than the costs) [44,45]. The employees who perceive equality and non-discrimination may be inspired to take voluntary actions to commit themselves on behalf of the firm. Samy et al. [46] point out that sustainability investment is a strategic move to maximize profits and fulfill various stakeholders’ demands. When employees perceive the safe environment and fair compensation provided by the organization, they are more likely to reciprocate and collaborate in return, as followed by the logic of social exchange theory. Therefore, EOO, through worker engagement, training, safety, and sustainability programs, have been shown to lower production costs and improve quality, thereby improving a firm’s financial performance [24,47]. Aggarwal [48] found that a firm’s governance rating controlled by employee-oriented performances has a significant positive impact on return on assets and equity. Firms with higher employee-oriented sustainable performance tend to outperform their competitors in terms of financial performance over time [49]. Hussain et al. [50] found that labor, decent work, and human rights positively correlate with accounting-based performances. From the SET perspective, improved equal opportunities for employees, quality of life and work, loyalty, diversification, and safety can lead to higher financial performance by increasing their commitment and cooperation with the firm, operating efficiency, and product quality. Thus, we predict:

**H5.**  *Employee-oriented outcomes have a positive direct relationship with firms’ financial performance.*

In addition to EOO, this study aims to analyze the direct impact of COO on firms’ financial performance. According to the resource-based view perspective, when a firm can control and obtain rare and non-substitutable resources, a sustainable competitive advantage is created by exploring new markets and developing new products [51,52,53]. Social investments of a firm, such as philanthropic activities and pollution prevention, may generate strong social value. Thus, they may be used as resources that create a competitive advantage [54]. In other words, these COO facilitate the firm to develop new opportunities in the uncertain market environment, thereby improving firms’ financial performance. Higher corporate social reputation levels and community contributions often lead to higher financial performance. In particular, a positive corporate image and reputation improve firm performance by attracting socially responsible investments and increasing customer satisfaction and sales [55,56]. Preston and O’bannon [57] proposed the three social dimensions of sustainability performance: employees, customers, and community. Their empirical results show a strong positive association between social and financial performance. Through a meta-analysis, Lu and Taylor [58] found that environmental and social sustainability positively affected financial performance. The market is expected to reward firms with high corporate sustainability performance in the long run. Lassala et al. [59] examined how corporate social performance relates to and leads to the final performance. They found a positive and causal relationship between communal aspects of sustainability outcomes and financial performance. A firm can benefit financially when markets and stakeholders recognize higher levels of its social responsibility, corporate reputation, public acceptance, and close ties with communities and NGOs on sustainability. Thus, we anticipate:

**H6.**  *Community-oriented outcomes have a positive direct relationship with firms’ financial performance.*

## 4. Experiments and Results

### 4.1. Data Collection and Measurements

Large-scale survey data were collected from USA manufacturing firms to test the hypotheses. We obtained the phone lists of the potential respondents from the Society of Manufacturing Engineers (SME). In total, 5000 phone lists from the SME were selected after carefully screening the SIC codes, job function, and job title, and then a telemarketing company was hired to collect data from the listed firms. Through these calls, a total of 531 respondents agreed to participate in the survey. Out of 531, 255 survey responses were received. After screening, 43 surveys were deleted from the database due to incomplete responses. The final number of usable responses was 212, with a response rate of 39.9%. About 65% of the firms were small and medium-sized companies (i.e., less than 250 employees), whereas 35% came from large organizations (i.e., larger than 250 employees). A quarter of the companies had annual revenues exceeding 100 million dollars, and approximately 61% had annual revenues less than 50 million dollars; in turn, 15% of the companies have revenue volumes between 51 and 100 million dollars. The sample covered firms under the two-digit codes SIC 30 (40.3%), SIC 34 (28.5%), SIC 35 (11.8%), SIC 36 (8.6%), SIC 37 (6%), and SIC 38 (4.8%). Survey responses were answered by the job titles of chief executive officer, chief operating officer, president, vice president, director, general manager, supply chain manager, or purchasing manager.

To measure the SCO, four items were adapted from Jaworski and Kohli [60], and Marz, Powers, and Queisser [16]. EWEP was measured by four items according to the work of Baptiste [61] and Wang [62]. Four items for CSIP were adapted from Valiente et al. [63] and Dey et al. [64]. EOO and COO were measured using five items adapted from Székely and Knirsch [65]. Lastly, financial performance was measured by three items adapted from Menor et al. [66]. A five-point Likert scale was used to measure all the constructs and the measurement items are presented in Appendix A.

### 4.2. Reliability and Validity

We conducted a confirmatory factor analysis to test the validity of our constructs using AMOS21. We found that our measurement model had acceptable fit indices: χ^2^/d.f. = 1.396, RMSEA = 0.043, CFI = 0.967, GFI = 0.884, NFI = 0.895 and IFI = 0.968. Table 1 shows all the measurement items, factor loadings, Cronbach’s alpha, compositive reliability, and average variance extracted (AVE) for each construct. All factors loaded ranged from 0.576 to 0.957, and the compositive reliability for each construct is greater than 0.737, indicating acceptable convergent validity. As shown in Table 2, the square root of the AVE for each construct was greater than the respective correlation coefficients, supporting the discriminant validity. The Cronbach’s alpha for the variables was greater than 0.7, which shows high reliability for our constructs.

### 4.3. Analysis Results

**Figure 1:**
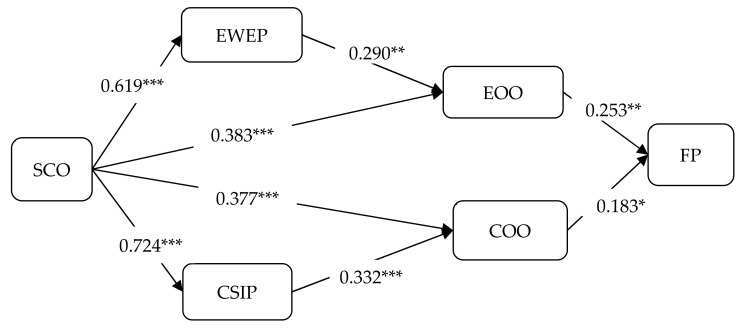
SEM results. SCO: social orientation of the firm; EWEP: employee well-being and equity practices; CSIP: corporate social involvement practices; EOO: employee-oriented outcomes; COO: community-oriented outcomes; FP: financial performance. * *p* < 0.05, ** *p* < 0.01, and *** *p* < 0.001.

We applied SEM to examine our hypothesized model by using AMOS21. Figure 1 illustrates our hypothesized model’s analysis results and standardized path estimates. The structural model presented acceptable model fit indices (χ^2^/d.f. = 1.535; RMSEA = 0.050; CFI = 0.957; GFI = 0.871; NFI = 0.886; IFI = 0.957). The results reveal that all relationships in our hypothesized model are significant and in accordance with our expectations.

H1a and H1b propose that the SCO is positively associated with EWEP and CSIP. The SEM results show that the SCO have significantly positive impacts on EWEP (path coefficient = 0.619, *p* < 0.001) and CSIP (path coefficient = 0.724, *p* < 0.001), supporting H1a and H1b. H2a and H2b proposed that the SCO is positively associated with EOO and COO. The results indicate that the SCO has significantly positive impacts on EOO (path coefficient = 0.383, *p* < 0.001) and COO (path coefficient = 0.377, *p* < 0.001), supporting H2a and H2b. H3, proposing that EWEP is positively related to EOO, is supported (path coefficient = 0.290, *p* < 0.01). H4, proposing that CSIP is positively related to COO, is supported (path coefficient = 0.332, *p* < 0.001). H5 and H6 predict that EOO and COO are positively associated with FP. Our results show that both EOO (path coefficient = 0.253, *p* < 0.01) and COO (path coefficient = 0.183, *p* < 0.05) have significantly positive impacts on FP, supporting H5 and H6.

## 5. Discussion

### 5.1. Theoretical Contributions and Managerial Implications

Based on social identity theory (SIT), social exchange theory (SET), and resource-based view (RBV), this study suggests a research model that explains how firms’ social orientation (i.e., highly employee-oriented and socially driven firms) would bring better social and financial performance. More specifically, the results of this study provide insights into the underlying mechanisms linking social orientation to social and financial performance via social sustainability practices (i.e., EWEP and CSIP). The results of this study contribute to the social sustainability literature by enriching our understanding of the important role of SCO in social sustainability practices. Our findings also suggest an influencing mechanism for transferring SCO into social sustainability performance and FP.

First, our results revealed that a firm’s SCO has a positive impact on EOO and COO directly. Similar to a previous study that emphasized the positive effect of social sustainability orientation on operational performance [34], our findings highlight that socially oriented firms bring better social sustainability performance, such as employee- and community-oriented performance outcomes. Second, in addition to these direct relationships, our findings reveal that a firm’s SCO indirectly facilitates social sustainability performance through social sustainability practices. Previous studies emphasize that a firm’s strategic orientation toward sustainability can promote sustainability practices [32,34]. In line with them, our findings reveal that a firm’s SCO facilitates EWEP and subsequently improves EOO. In addition, a firm’s SCO facilitates CSIP and then improves the COO. Likewise, our findings highlight that a firm’s SCO influences social sustainability performance (i.e., EOO and COO) through the different social sustainability practices (i.e., EWEP and CSIP). Lastly, EOO and COO have positive impacts on FP. Based on SET and RBV, both internal (employee) and external (community and society) sustainability performance outcomes can bring employee dedication and social recognition, thereby increasing financial returns.

Our research also has important implications for managers. Financial performance improvement is a key driver motivating enterprises to embrace social-orientation practices in their operations. Our findings support the essential role of SCO in promoting social sustainability practices and improving performance outcomes. According to our findings, firms’ executives should be cognizant of the impact mechanisms in which SCO works to benefit FP. Specifically, they must adopt different approaches for improving different types of social sustainability performance. For instance, their strategic effort on SCO can improve EOO directly and indirectly via EWEP. Thus, they need to recognize the importance of implementing EWEP, such as providing a safe work environment, fair compensation, and gender equality for employees, especially when they attempt to improve EOO and FP by applying SCO. In addition, their effort on SCO not only improves the COO directly but also does so indirectly via CSIP. Therefore, firms’ executives should pay close attention to implementing CSIP to increase the benefits of their SCO in improving COO and FP. In this way, our findings provide valuable insights into how the executives’ efforts on SCO can improve EOO and COO more efficiently by leveraging EWEP and CSIP, respectively.

### 5.2. Limitations and Future Research Directions

Although this study provides theoretical and managerial implications, it is not free of limitations. First, our samples are limited to US manufacturing companies. Since the role of SCO in social sustainability practices may vary under the different contingency factors, such as cultural background and economic development status, future research could be conducted to further investigate such factors’ contingent role by collecting data from multiple other countries. Second, although this study introduced CSIP as an external social sustainability practice by focusing on firms’ philanthropic commitment within a community and to a larger society, its theoretical background can be further developed to expand our understanding of how to design and implement active social involvement practices. Third, future studies could adopt a different research design (e.g., a longitudinal case study), which provides the practical basis for the firms’ short-term, medium-term, and long-term financial performance development while implementing social sustainability practices.

## 6. Conclusions

This study investigates the antecedents and output performance of social sustainability practices. The results obtained from the empirical study show that a firm’s SCO plays a very important role in promoting the two dimensions of social sustainability practices (i.e., EWEP and CSIP) that further influence social sustainability performance (i.e., EOO and COO). More specifically, SCO can improve EOO via EWEP and COO via CSIP. The results also reveal that social sustainability performance can lead to the better financial performance of firms. By providing a research model that explains the influencing mechanisms linking SCO to performance outcomes, this study contributes to social sustainability research.

## Figures and Tables

**Table 1:** Measurement model item loadings and reliability.

Constructs (Variables)	Measurement Item	Factor Loadings	Cronbach’s α	CR	AVE
Social Orientation (SCO)	SCO1	0.640	0.885	0.870	0.629
	SCO3	0.884			
	SCO4	0.806			
	SCO5	0.822			
Employee well-being and equity practices (EWEP)	EWEP2	0.576	0.700	0.737	0.415
	EWEP3	0.613			
	EWEP4	0.598			
	EWEP5	0.771			
Corporate social involvement practices (CSIP)	CSIP1	0.848	0.923	0.928	0.762
	CSIP2	0.878			
	CSIP4	0.880			
	CSIP5	0.886			
Employee-oriented outcomes (EOO)	EOO1	0.742	0.849	0.867	0.547
	EOO2	0.790			
	EOO3	0.734			
	EOO4	0.616			
	EOO5	0.802			
Community-oriented outcomes (COO)	COO1	0.685	0.865	0.870	0.575
	COO2	0.842			
	COO3	0.758			
	COO6	0.807			
	COO7	0.685			
Financial Performance (FP)	FP1	0.795	0.923	0.928	0.813
	FP2	0.957			
	FP3	0.944			

**Table 2:** Inter-construct correlations and AVEs.

	Mean	S.D.	1	2	3	4	5	6
1.Firm size	2.264	1.354						
2.SCO	3.434	0.751	0.029					
3.EWEP	3.730	0.781	−0.020	0453 **				
4.CSIP	2.814	1.157	0.052	0.652 **	0.330 **			
5.EOO	3.412	0.504	0.004	0.518 **	0.402 **	0.376 **		
6.COO	3.274	0.446	0.027	0.535 **	0.239 **	0.536 **	0.493 **	
7.FP	3.512	0.790	0.025	0.293 **	0.160 *	0.171 *	0.317 **	0.285 **

Note: *n* = 212; * *p* < 0.05, ** *p* < 0.01.

## Data Availability

Not applicable.

## Appendix A. Survey Items

Social Orientation (SCO)	
SCO1	Our firm’s mission statement communicates the importance of employees’ well-being.
SCO3	Our firm is committed to enhancing social responsibility.
SCO4	Our employees understand the importance of social responsibility.
SCO5	Our firm evaluates social implications of our operational decisions.
Employee Well-being and Equity Practices (EWEP)	
EWEP2	Our firm is committed to safe work environment.
EWEP3	Our firm’s management is quite culturally diverse.
EWEP4	Our firm provides fair compensation.
EWEP5	Our senior management reflects gender equality.
Corporate Social Involvement Practices (CSIP)	
CSIP1	Our firm contributes to charitable causes
CSIP2	Our firm volunteers for social causes.
CSIP4	Our firm has volunteers supporting local charities.
CSIP5	Our firm donates to community organizations.
Employee-Oriented Outcomes (EOO)	
EOO1	Employee quality of life.
EOO2	Employee health and safety
EOO3	Employee fair compensation.
EOO4	Fair employment opportunity
EOO5	Employment gender equality
Community-Oriented Outcomes (COO)	
COO1	Corporate reputation.
COO2	Social commitment.
COO3	Reportable contributions to communities.
COO6	The relationship with local communities
COO7	The relationship with NGOs
Financial Performance (FP)	
FP1	Return on investment (ROI).
FP2	Return on asset (ROA).
FP3	Profit margin on sales.
